# Disseminated Kaposi Sarcoma With Ocular Involvement: An Unusual Multisystem Case Presentation

**DOI:** 10.7759/cureus.90070

**Published:** 2025-08-14

**Authors:** Ana de Lourdes Torralbas Fitz, Idania Maria Cruzata Matos, Sergio Jose Torralbas Fitz, Eliany Leon Figueredo, Elizabeth Blanco Espinosa

**Affiliations:** 1 Oncology, Maputo Central Hospital, Maputo, MOZ; 2 General Medicine, Facultad de Ciencias Medicas, Holguin, CUB; 3 Miller School of Medicine, University of Miami, Miami, USA; 4 General Medicine, Englewood Health Physician Network - Primary Care, Englewood, USA; 5 Surgery, Hospital Arnaldo Milian, Miami, USA

**Keywords:** antiretroviral therapy, chemotherapy, hiv/aids, kaposi sarcoma, liposomal doxorubicin, ocular involvement

## Abstract

Kaposi sarcoma (KS) is an angioproliferative malignancy associated with human herpesvirus 8 (HHV-8) infection, predominantly affecting immunocompromised patients such as those with HIV/AIDS. Despite advances in antiretroviral therapy, KS remains a significant cause of morbidity and mortality in this population, especially when diagnosis or treatment is delayed. Ocular involvement, although rare, can lead to significant functional impairment. We report the case of a 21-year-old HIV-positive female on antiretroviral therapy who presented with extensive cutaneous lesions, facial infiltration, periorbital edema causing visual obstruction, and cervical lymphadenopathy. Histopathologic examination confirmed nodular-stage KS. The patient was staged as T1 I1 S1 according to the AIDS Clinical Trials Group (ACTG) classification, indicating advanced disease. Treatment included continuation of antiretroviral therapy, blood transfusions, and systemic chemotherapy with liposomal doxorubicin. After four cycles, she achieved complete clinical and immunologic remission, with resolution of lesions and restoration of visual function. This case highlights the critical role of early recognition, histopathologic diagnosis, and multidisciplinary management in HIV-associated KS. It also underscores the potential for favorable outcomes with timely and integrated therapy, even in advanced cases with ocular involvement.

## Introduction

Kaposi sarcoma (KS) is a rare angioproliferative tumor caused by human herpesvirus 8 (HHV-8), primarily affecting the skin and mucous membranes, including the oral cavity and other mucosal sites. It also involves regional lymph nodes and may disseminate to various internal organs such as the lungs and liver. In advanced or disseminated disease, KS frequently affects the gastrointestinal system, presenting with lesions throughout the mucosal lining of the digestive tract, which can lead to symptoms such as bleeding or obstruction. These internal organ involvements contribute to morbidity, particularly among immunosuppressed individuals, especially those living with HIV. Although HHV-8 infection is necessary for KS development, additional genetic, environmental, and immunologic factors contribute to its pathogenesis. Immunosuppression, particularly due to HIV infection, significantly elevates the risk of KS, making it one of the most common malignancies among people living with HIV (PLWH). Four epidemiologic forms of KS have been described: classic KS, typically occurring in older adults of Mediterranean or Eastern European descent without HIV; iatrogenic KS, seen in transplant recipients undergoing immunosuppressive therapy; endemic KS in parts of Africa; and epidemic KS associated with HIV infection [1].

In the United States, KS incidence peaked in the late 1980s at approximately 33 cases per 100,000 person-years, coinciding with the HIV/AIDS epidemic, and subsequently declined following the widespread adoption of combination antiretroviral therapy (ART) in the late 1990s. Despite this decline, disparities remain: African Americans, who accounted for only 12% of the US population in 2014, represented 50% of new HIV diagnoses. Geographic variations in HIV testing, treatment access, and mortality persist, especially in southern states. Recent data show a shift in KS incidence towards younger age groups (25-34 years) after 2015, with persistently higher rates among males, elderly adults, and Black/African American populations. States such as California, New York, and Texas exhibit the highest burden of KS; each of these states reports between 5,000 and 10,000 cases, corresponding to crude rates ranging from 0.5 to one per 100,000 population; whereas others, such as Idaho, Montana, and South Dakota, reported the lowest case counts and crude incidence rates nationwide [2,3]. These disparities in HIV diagnosis and healthcare access contribute to delays in KS screening and diagnosis, resulting in more advanced disease at presentation and increased morbidity and mortality among affected populations.

KS most commonly affects the skin, especially on the face, trunk, and upper and lower limbs, presenting as characteristic purplish or brownish lesions. Mucosal involvement - including the oral cavity, oropharyngeal region, and genital mucosa - occurs in about 20% of patients. In its disseminated form, KS can spread beyond the skin to internal organs such as the lungs, gastrointestinal tract, and lymph nodes, often resulting in significant morbidity and complex clinical challenges [4].

Ongoing surveillance is crucial for informing effective cancer control strategies, reducing healthcare disparities, and enhancing outcomes in vulnerable populations. In this report, we describe an unusual presentation of KS, aiming to emphasize the wide spectrum of clinical manifestations and to raise awareness among healthcare providers.

## Case presentation

A 21-year-old female patient, known to be HIV-positive and on ART consisting of tenofovir disoproxil fumarate (TDF) 300 mg/24 hours, lamivudine (3TC) 300 mg/24 hours, and dolutegravir (DTG) 50 mg/24 hours, was referred from the health center with clinical suspicion of KS. On physical examination, multiple hyperpigmented cutaneous lesions were noted on the lower limbs, the largest measuring approximately 4 cm in diameter, along with smaller lesions on the face, each around 2 cm.

**Figure 1:**
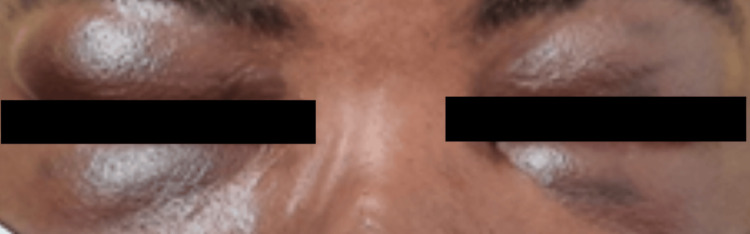
Significant facial infiltrate with marked periorbital edema.

Palpation revealed bilateral cervical lymphadenopathy at levels III and IV, with nodes approximately 2 cm in size. A significant facial infiltrate with marked periorbital and palpebral edema (+++) was present (Figure 1), leading to substantial limitation in daily activities as a result of impaired ocular opening.

**Figure 2:**
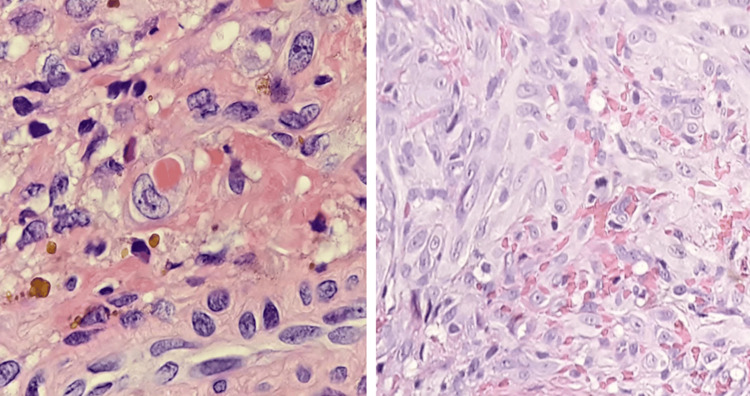
Spindle cells and hemosiderin deposits, characteristics of Kaposi sarcoma.

Laboratory investigations demonstrated severe anemia (Hb: 7 g/dL), leukopenia (WBC: 3 x 10⁹/L) with relative lymphocytosis (73%), preserved platelet count (230 x 10⁹/L), and a markedly reduced CD4+ T-cell count (89 cells/mL). Imaging studies did not reveal additional findings. The Karnofsky Performance Status was assessed at 50%. Histopathologic examination of a cutaneous lesion confirmed the diagnosis of KS, showing classic features of nodular-stage disease such as interlacing fascicles of spindle cells, slit-like vascular spaces with extravasated erythrocytes, hemosiderin-laden macrophages, and minimal nuclear atypia (Figure 2). Based on these findings, the patient was staged as WHO HIV Clinical Stage IV with KS (T1I1S1) according to the AIDS Clinical Trials Group (ACTG) staging criteria [5].

**Figure 3:**
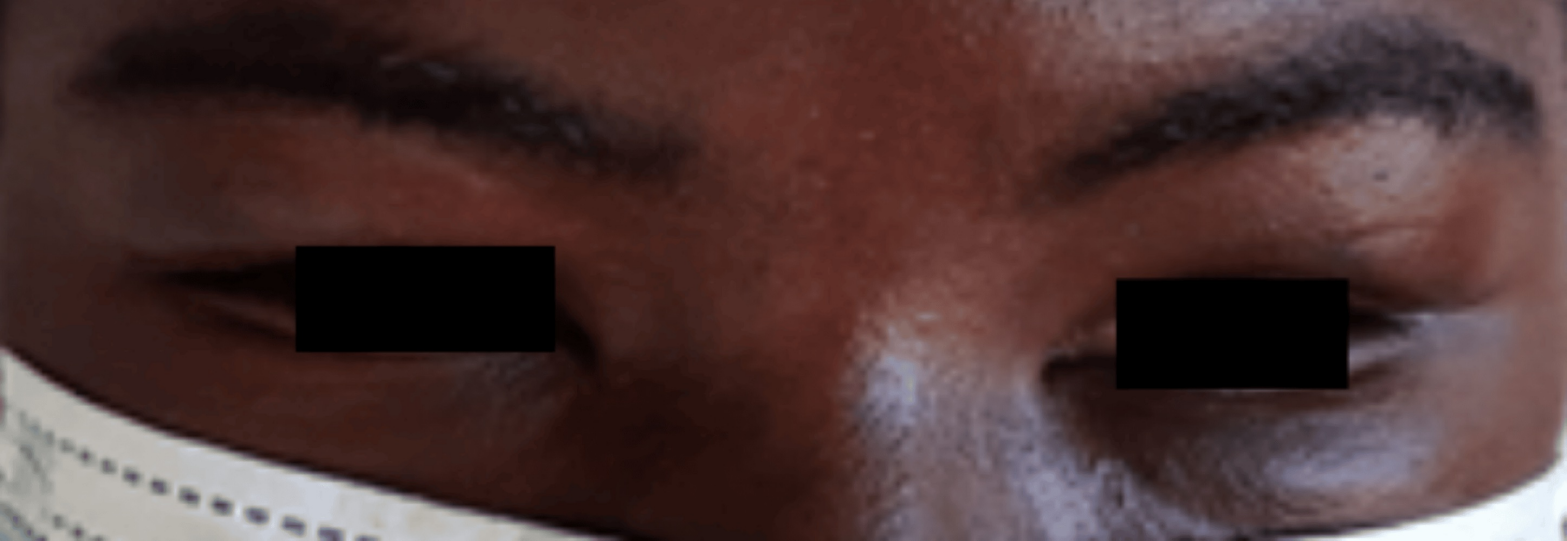
Significant recovery after treatment.

Management included transfusion of two units of packed red blood cells, continuation of ART, and initiation of chemotherapy with liposomal doxorubicin at a dose of 20 mg/m² every 21 days. After four cycles of chemotherapy, the patient achieved a complete clinical response (Figure 3), with total resolution of skin lesions, regression of cervical lymphadenopathy, and full remission of facial and periorbital edema, resulting in complete restoration of visual function. Post treatment, immunologic recovery was demonstrated by an increase in the CD4+ count to 568 cells/mL.

## Discussion

KS was the first malignancy recognized as an AIDS-defining illness in 1981 and remains the most common AIDS-associated malignancy in sub-Saharan Africa. It is a multicentric, highly vascularized tumor characterized by the proliferation of spindle cells accompanied by inflammatory infiltration of monocytes, T lymphocytes, and plasma cells. Clinically, KS typically presents as violaceous or brownish papules and nodules that may coalesce into plaques. These lesions frequently involve the face, trunk, and extremities. Mucosal involvement - including the oral cavity, oropharyngeal region, and genital mucosa - occurs in approximately 20% of cases. In advanced stages, KS may extend beyond the skin to internal organs such as the lungs, gastrointestinal tract, lymph nodes, and ocular structures, often resulting in significant morbidity [6].

Although KS predominantly occurs in HIV-positive individuals, cases in HIV-negative patients without known immunodeficiency have also been reported. Such presentations, though uncommon, are clinically significant as they broaden the recognized spectrum of KS and underscore the need to consider it in the differential diagnosis, even in HIV-negative individuals, particularly in the presence of HHV-8 infection and atypical features [7].

In contrast, the patient presented in this report is an HIV-positive female on ART, illustrating the classic association between KS and AIDS. She exhibited multiple hyperpigmented cutaneous lesions on the lower limbs and face, bilateral cervical lymphadenopathy, and a marked facial infiltrate with periorbital edema resulting in complete visual obstruction. Ocular involvement occurs in about 20% of AIDS-related KS cases, with conjunctival lesions being the initial manifestation in roughly 12%. Most ocular lesions grow slowly and are often located in the inferior fornix, where they can be managed effectively with simple surgical excision or adjunctive cryotherapy. Radiotherapy and chemotherapy are generally reserved for aggressive or multifocal disease presentations [8].

Biopsy is essential to confirm KS diagnosis, whether the lesions are conjunctival, periocular, cutaneous, or visceral. Histopathologically, the hallmark features include proliferation of spindle-shaped endothelial cells forming slit-like vascular channels dissecting through collagen bundles, with frequent extravasation of erythrocytes and hemosiderin deposits. The lesions have been categorized into three types based on their histopathological features: type I features dilated vascular channels lined by flat endothelial cells; type II exhibits larger spindle cells with hyperchromatic nuclei; and type III shows dense spindle cells with occasional mitoses. These subtypes represent a pathological continuum and may coexist within the same lesion [9]. Immunohistochemical analysis typically demonstrates positivity for endothelial and lymphatic markers, such as CD31, CD34, factor VIII-related antigen, podoplanin (D2-40), and factor VII. Detection of HHV-8 DNA or latent nuclear antigen LANA-1 is crucial for diagnosis and to distinguish KS from other vascular tumors. While several histologic variants exist, traditional TNM (tumor/node/metastasis) staging is not well-suited for KS due to its cutaneous predominance and indolent progression. Instead, staging systems such as the ACTG classification are preferred [10].

Following histopathologic confirmation, staging becomes essential to guide prognosis and management. The ACTG TIS system considers tumor burden (T), immune status (I), and systemic illness (S). In this case, the patient was staged as T1 I1 S1. She met the criteria for T1 due to extensive cutaneous lesions and lower extremity edema; I1 due to a CD4 count below 200 cells/mm³; and S1 based on systemic symptoms such as weight loss and signs of opportunistic infection. This classification indicates a poor prognosis and supports the need for systemic chemotherapy in addition to ART [5,11].

Histologic examination of the patient’s biopsy revealed a densely cellular dermal proliferation of spindle-shaped cells in interlacing fascicles, with mildly pleomorphic nuclei and vesicular chromatin. Numerous slit-like vascular spaces containing extravasated erythrocytes were noted, along with hemosiderin-laden macrophages and occasional binucleated spindle cells. No significant atypia or abnormal mitoses were present, consistent with nodular-stage KS rather than a high-grade sarcomatoid neoplasm.

These features are consistent with the classical histological presentation of KS described in the literature. It is commonly noted that there is a proliferation of spindle-shaped endothelial cells forming narrow vascular spaces that infiltrate collagen bundles, frequently accompanied by extravasated red blood cells and hemosiderin accumulation. The presence of spindle cell fascicles, erythrocyte extravasation, and hemosiderin deposits is recognized as characteristic of the nodular form of the disease [12-15].

The cornerstone of systemic treatment for all KS subtypes is the optimization of immune function and suppression of HIV to limit disease progression. In AIDS-related KS, ART is essential, as viral suppression supports immune recovery and limits HHV-8 activity. First-line chemotherapeutic options include liposomal doxorubicin and paclitaxel. While both are effective, paclitaxel may present higher toxicity. For classic KS, retrospective series suggest that liposomal doxorubicin, paclitaxel, and low-dose interferon offer favorable outcomes. In relapsed or refractory cases, agents such as pomalidomide, bortezomib, gemcitabine, or lenalidomide may be considered depending on prior response [16].

In line with these guidelines, our patient received transfusions, continued ART, and began liposomal doxorubicin at 20 mg/m² every 21 days. She achieved complete clinical response after four cycles, with full resolution of lesions and restoration of visual function, accompanied by significant immunologic improvement.

This case illustrates the classical clinical, histopathologic, and therapeutic features of AIDS-related KS. It emphasizes the importance of early clinical suspicion in patients with rapidly progressive violaceous lesions, prompt biopsy for diagnosis, and a multidisciplinary approach to treatment. Timely initiation of ART and systemic chemotherapy can lead to full clinical and immunological recovery, even in patients presenting with advanced-stage disease.

## Conclusions

This case highlights the importance of maintaining a high index of suspicion for disseminated KS in immunocompromised patients, particularly when ocular or craniofacial manifestations are present. Although rare, ocular involvement may serve as an early and visually significant indicator of disease. Timely diagnosis and targeted treatment are essential to preserve vision and reduce systemic burden. Routine ophthalmologic assessment should be considered in HIV-positive individuals with facial lesions.

Additionally, this report emphasizes the relevance of KS in women, a group often underrepresented in clinical research. The positive outcome achieved through combined antiretroviral therapy and liposomal chemotherapy supports the effectiveness of integrated HIV-oncology care. Even in advanced-stage disease, complete remission is achievable with early and coordinated intervention. Continuous clinical, immunologic, and virologic follow-up is vital to optimize long-term outcomes.
